# Role of Artificial Intelligence in MRI-Based Rectal Cancer Staging: A Systematic Review

**DOI:** 10.7759/cureus.76185

**Published:** 2024-12-22

**Authors:** Afsal Latheef Tayyil Purayil, Rahul M Joseph, Arjun Raj, Aswathy Kooriyattil, Nihala Jabeen, Saima Fazila Beevi, Najiyah Lathief, Fasil Latheif

**Affiliations:** 1 Academy of Surgery, Barking, Havering and Redbridge University Hospitals NHS Trust, London, GBR; 2 Emergency Medicine, Government Tirumala Devasom Medical College, Alappuzha, Alappuzha, IND; 3 Internal Medicine, King's College Hospital NHS Foundation Trust, London, GBR; 4 General Medicine, King's College Hospital NHS Foundation Trust, London, GBR; 5 Unani Medicine, Markaz Unani Medical College and Hospital, Kozhikode, IND; 6 Dentistry, PMS College of Dental Science and Research, Trivandrum, IND; 7 Medicine, Batterjee Medical College, Jeddah, SAU; 8 Internal Medicine, Belgaum Institute of Medical Science, Belgaum, IND

**Keywords:** artificial intelligence, deep learning, diagnosis, magnetic resonance imaging, rectal cancer

## Abstract

Several studies explored the application of artificial intelligence (AI) in magnetic resonance imaging (MRI)-based rectal cancer (RC) staging, but a comprehensive evaluation remains lacking. This systematic review aims to review the performance of AI models in MRI-based RC staging. PubMed and Embase were searched from the inception of the database till October 2024 without any language and year restrictions. The prospective or retrospective studies evaluating AI models (including machine learning (ML) and deep learning (DL)) for diagnostic performance in MRI-based RC staging compared with any comparator were included in this review. The performance metrics were considered as outcomes. Two independent reviewers were involved in the study selection and data extraction to limit bias; any disagreements were resolved through mutual consensus or discussion with a third reviewer. A total of 716 records were identified from the databases. Out of these, 14 studies (1.95%) were finally included in this review. These studies were published between 2019 and 2024. Various MRI technologies were adapted by the studies and multiple AI models were developed. DL was the most common. The MRI images including T1-weighted images (14.28%), T2-weighted images (85.71%), diffusion-weighted images (42.85%), or the combination of these from different landscapes and systems were used to develop the AI models. The models were built using various techniques, mainly DL such as conventional neural network (28.57%), DL reconstruction (14.28%), Weakly supervISed model DevelOpment fraMework (7.12%), deep neural network (7.12%), Faster region-based CNN (7.12%), ResNet, DL-based clinical-radiomics nomogram (7.12%), LASSO (7.12%), and random forest classifier (7.12%). All the models that used single-type images or combined imaging modalities showed a better performance than manual assessment in terms of higher accuracy, sensitivity, specificity, positive likelihood ratio, negative likelihood ratio, and area under the curve with a score of >0.75. This is considered to be a good performance. The current study indicates that MRI-based AI models for RC staging show great promise with a high performance.

## Introduction and background

Rectal cancer (RC) is one of the leading causes of cancer-related morbidity and mortality worldwide [1]. As per the recent Global Burden of Disease Study, colon and RC accounted for an age-standardised incidence rate of 25.61%, being third in countries with higher human development index [2]. Accurate staging of RC is critical for treatment planning, determining prognosis, and evaluating therapeutic responses [1]. Magnetic resonance imaging (MRI) has become the gold standard for preoperative staging of RC due to its high soft tissue contrast and ability to assess tumour extent, lymph node involvement, and vascular invasion [3,4]. Despite advancements in MRI technology, the assessment of RC remains subject to interobserver variability and can be time-consuming, requiring highly trained radiologists to interpret complex imaging features [5].

RC staging is crucial for determining prognosis and guiding treatment decisions, with the TNM (tumour, node, metastasis) system being a widely accepted, guideline-recommended and globally used method. It incorporates tumour size, local invasion, lymph node involvement, and distant metastasis, which collectively inform the clinical approach. It also allows the healthcare practitioner to decide the best possible treatment approach as per patient requirements [6,7]. Imaging techniques like CT, MRI, and ultrasound play significant roles in staging, with CT being the gold standard for assessing tumour size, extension, and metastasis [8]. The integration of artificial intelligence (AI) in RC staging shows great promise, particularly in enhancing the accuracy of image interpretation, automating the detection, and segmentation of tumours, improving the precision of radiological staging by reducing human error and providing deeper insights into tumour behaviour [9]. Recent studies suggest that AI-based analysis of CT and MRI scans, coupled with data from molecular biomarkers, offers the greatest opportunity to improve accuracy and clinical outcomes in renal cancer staging [10].

In recent years, AI, particularly deep learning (DL) algorithms, has shown significant promise in improving the accuracy and efficiency of medical imaging analysis [11,12]. AI models have the potential to automate the interpretation of MRI images, reduce human error, and provide more reliable prognostic predictions. Several studies have explored the application of AI in MRI-based RC staging, demonstrating promising results in terms of diagnostic accuracy, sensitivity, and specificity [13,14]. However, to date, a comprehensive evaluation of the prognostic performance of AI models in this context remains lacking.

This systematic review aims to assess the current evidence on the diagnostic performance of AI models in MRI-based staging of RC, focusing on their accuracy, sensitivity, specificity, and overall performance in staging the RC.

## Review

Methodology

We followed the Preferred Reporting Items for Systematic Reviews and Meta-analysis (PRISMA) to report this study [15,16]. We followed the Participants, Intervention, Comparator, Outcomes and Study design (PICOS) framework to include the studies in this review. The protocol for this systematic review is registered in PROSPERO with a registration number CRD42024615875.

Search Strategy

A comprehensive search of the literature was conducted in PubMed and Embase from Inception till October 2024 without any restriction to language and year. The search strategy incorporated all the possible key terms and their combinations: “artificial intelligence”, “rectal cancer”, and “MRI”. The recent cancer conference abstract databases from the American Society of Clinical Oncology and American Association for Cancer Research were searched for any relevant studies. In addition, the reference lists of relevant articles were also hand-searched to identify additional studies. A detailed search strategy is provided in Appendix A.

Inclusion and Exclusion Criteria

Studies were included in the review if they met the following criteria: The prospective or retrospective studies that evaluated AI models (including ML and DL) for diagnostic performance in MRI-based RC staging. The studies should include adult patients diagnosed with RC irrespective of their operative status. We considered the conventional methods, manual methods, human interpretation, non-AI-based MRI interpretation, or any other comparisons in this review. The performance metrics such as sensitivity, specificity, accuracy, positive predictive value (PPV), negative predictive value (NPV), positive likelihood ratio (PLR), negative likelihood ratio (NLR), area under the curve (AUC), and C-index were considered as the outcome. Studies focusing on non-AI-based models, any other purpose not of staging, non-English language studies, and studies that did not report the performance metrics were excluded (see Appendix B).

Study Selection

The retrieved studies from the databases were added to Microsoft Excel (Microsoft Corporation, USA) and duplicates were removed. The titles and abstracts of the remaining studies were screened in accordance with pre-defined criteria. The full-texts of the selected studies were downloaded and screened using the same criteria and studies that passed this stage were considered for final inclusion in our review. Two independent reviewers (AL and AR) were involved in the study selection to limit bias; any disagreements were resolved through mutual consensus or discussion with a third reviewer (AK).

Data Extraction

Data from the included studies were independently extracted by two reviewers (AL, RMJ) using a standardized data extraction form. Extracted data includes the study characteristics, AI model characteristics, outcome measures, and key model performance. We did not perform the quality assessment as there are no specific tools to assess the risk of bias in AI model studies. Any discrepancies in data extraction are settled through mutual discussion or consultation with another reviewer (NJ).

Data Synthesis

Data synthesis was conducted descriptively due to the heterogeneity of the included studies in terms of AI models, MRI techniques, and outcome measures. A narrative summary of the prognostic performance of AI models in MRI-based RC staging was provided, with a focus on the most commonly reported outcomes. No meta-analysis was performed due to the highly heterogeneous nature of the studies.

Results

Study Selection

**Figure 1:**
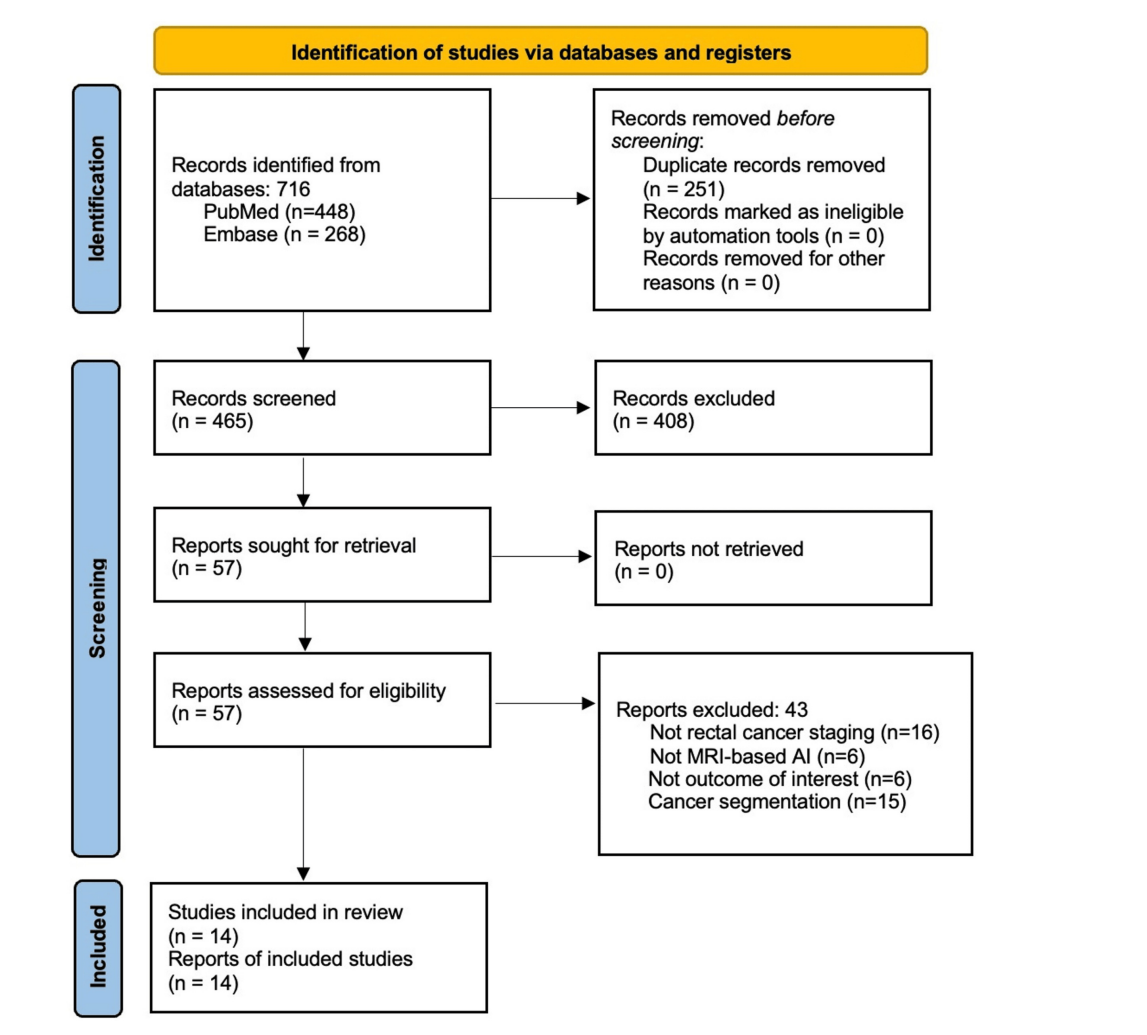
Preferred Reporting Items for Systematic Reviews and Meta-analysis (PRISMA) flow diagram for the study selection

A total of 716 records were identified from the databases, out of which 465 articles were screened for their title and abstracts after duplicate removal. Among them, 57 full-text articles were assessed for eligibility and 14 (1.95%) studies [17-30] were finally included in this review (Figure 1).

Study Characteristics

A total of 14 studies [17-30] published between 2019 and 2024 were included in this review. Among the included studies, 12 studies (85.7%) considered retrospective data to build the model, whereas the remaining two studies (14.3%) used prospective data. The majority (n = 10, 71.4%) were single-centre studies, whereas four studies (28.6%) used the data from multicentric settings. The detailed study characteristics are provided in Table 1.

**Table 1:** Study characteristics and AI model specifications AUC: area under the curve; CCCD: Chinese Colorectal Cancer Database; CNN: conventional neural network; DL: deep learning; DLR: deep learning reconstruction; DLRS: deep learning radiomic signature; DNN: deep neural network; DWI: diffusion-weighted image; LARC: locally advanced rectal cancer; LASSO: least absolute shrinkage and selection operator; NR: not reported; RC: rectal cancer; RF: random forest; ResNet: residual networks; SR: super-resolution; T1WI: T1-weighted imaging; T2WI: T2-weighted and diffusion-weighted imaging; WISDOM: Weakly supervISed model DevelOpment fraMework model; 2D: two dimensional; 3D: three dimensional

Author and year	Country	Purpose of AI	Source of data	Type of data	Type of AI and comparator	Performance Metrics
Xia W et al., 2024 [30]	China	Binary and ternary N staging in RC patients	Retrospective data from three hospitals (Jan 2016-Nov 2017)	T2WI- MRI images	WISDOM vs. senior and junior radiologists	AUC, C-index
Hamabe A et al., 2024 [25]	China	mrAI-based T-stage prediction of RC	Retrospective data from 69 institutions, (Jan 2010-Dec 2011)	MRI	MRI-assisted AI-based algorithm vs. radiologist and pathologist	Sensitivity, specificity, accuracy
Peng W et al., 2024 [28]	China	Performance of DLR-based FSE MRI-assisted T and N staging in RC	Prospective data from a single hospital (Nov 2022 - May 2023)	T2WI	DLR-based FSE MRI vs. standard FSE MRI	T-staging accuracy
Tian C et al., 2023 [23]	China	Differentiate between different T-stages of RC	Retrospective data of a single hospital (Jan 2017 - May 2023)	HR-T2W images	Deep learning and CNN vs. subjective evaluation	AUC, sensitivity, specificity, accuracy, PLR, NLR, PPV, NPV, NRI
Wan L et al., 2023 [19]	China	Lymph node metastasis for the predication in stage T1-2 RC patients	Retrospective study (Oct 2013 -Mar 2021)	T2WI	Four 2D- and 3D-based ResNet (ResNet18, ResNet50, ResNet101, and ResNet152) vs. Radiologist	AUC
Ma S et al., 2023 [20]	China	N-stage prediction among RC patients	Retrospective trial (Jan 2017 -Jan 2020)	Axial DWI, T2WI, T1WI and HR-T2WI	DL-based clinical-radiomics nomogram vs. subjective evaluation and Radscore	AUC, sensitivity, specificity, accuracy, PLR, NLR, PPV, NPV
Wei Y et al., 2023 [21]	China	DL model for improving T-staging accuracy in RC patients	Retrospective study, (NR)	Dynamic contrast-enhanced T2WI and DWI	DL model of multiparametric CNN vs. clinical model and radiologist assessment	AUC
Hou M et al., 2022 [18]	China	DL-based 3D-SR MRI radiomics model for preoperative T-staging in RC	Retrospective study (Jan 2014 to Oct 2020)	Combination of oblique axial HRT2WI, sagittal T2WI, axial DWI, axial T1WI, and gadolinium contrast-enhanced T1WI of the pelvis	DL-3D SR vs. expert radiologists	Accuracy, sensitivity, specificity, and AUC
Yuan X et al, 2022 [22]	China	DL model for improving T-staging accuracy in RC patients	Prospective study (NR)	HR-T2WI	LASSO with vs. without rectal filling	AUC
Liu X et al., 2021 [17]	China	Prediction of metastasis-based T-staging in LARC patients	Retrospective data from three hospitals (Aug 2012-Mar 2015)	Multiparametric MRI, T2WI, DWI	DLRS vs. manual	C-index, NRI, IDI
Wu QY et al., 2021 [26]	China	Predicting preoperative T-staging	Retrospective data from CCCD (Jul 2016-Jul 2017)	MRI	DNN (Faster region-based CNN) vs. manual	AUC, accuracy
Wen X et al., 2021 [29]	China	DL model for predicting preoperative T-staging in RC patients	Retrospective study in a single centre (Oct 2017 -Dec 2018)	T2WI and RS-EPI DWI images	Automated ML model vs. conventional method	Sensitivity, specificity, AUC, Accuracy
Zhao X et al., 2019 [24]	China	Lymph node detection and N-staging in RC	Retrospective data from 6 hospitals (Jul 2013-June 2016)	Fused T2WI and DWI	Deep learning framework Mask R-CNN vs. radiologists	Sensitivity, PPV, FP/Vol, DSC
He B et al., 2019 [27]	China	MRI‐based tumour grading of RC using RF model	Retrospective study in single centre (NR)	Axial T2‐weighted turbo spin echo sequence with no fat suppression	Radiomics-based random forest classifier models	AUC, sensitivity, specificity

AI Model Characteristics

The MRI images including T1-weighted images (T1WI, 14.28%), T2-weighted images (T2WI, 85.71%), diffusion-weighted images (DWI, 42.85%), or the combination of these from different landscapes and systems were used to develop the AI models. The models were built using various techniques, mainly DL such as conventional neural network (CNN, 28.57%), DL reconstruction (DLR, 14.28%), Weakly supervISed model DevelOpment fraMework (WISDOM, 7.12%), deep neural network (DNN, 7.12%), Faster region-based CNN (7.12%), ResNet, DL based clinical-radiomics nomogram (7.12%), LASSO (7.12%), and random forest classifier (7.12%). The outcomes such as AUC, sensitivity, specificity, accuracy, PLR, NLR, PPV, and NPV along with C-index were majorly assessed in the included studies. The information on the AI model is presented in Table 1. The studies used about 70-80% of the data to train the model and 30-20% to test the model. However, the studies by Xia et al. [29] and Ma S et al. [19] used only 60% of their data to train the AI model (Table 2).

**Table 2:** Performance of AI models in MRI-based rectal cancer staging AUC: area under the curve; CI: confidence interval; DCE: dynamic contrast enhanced; NLR: negative likelihood ratio; NPV: negative predictive value; NR: not reported; NRI: net classification index; PLR: positive likelihood ratio; PPV: positive predictive value; SE: standard error

Author and year	Number of patients	Training set (%)	Test set	Performance of AI	Conclusion
Xia W et al., 2024 [30]	1014	589 (58.1)	146 (14.4)	AUC MI: 0.78 (95% CI: 0.73, 0.82); MIS: 0.80 (95% CI: 0.76, 0.84; P = 0.006); MISA: 0.81 (95% CI: 0.77, 0.86; P = 0.04); Junior radiologist: 0.69 (95%CI: 0.64, 0.73); Junior radiologist + AI: 0.80 (95%CI: 0.76, 0.84; P<0.001); Senior radiologist: 79.2 (75.1, 83.0); Senior radiologist + AI: 0.88 (0.85, 0.91; P<0.001) C-Index MI: 0.73 (95% CI: 0.69, 0.77); MIS: 0.74 (95% CI: 0.70, 0.78; P = 0.02); MISA: 0.76 (95% CI: 0.73, 0.80; p = 0.03); Improvement in senior radiologist + AI vs. senior radiologist (p = 0.007); Improvement in junior radiologist + AI vs. junior radiologist (p = 0.001)	The WISDOM model better performed than the human radiologists. A better performance was observed when combining AI with human.
Hamabe A et al., 2024 [25]	467	NR	NR	Centralized diagnosis Sens: 84.2; Spec: 37.7; Acc: 73.7 mrAI diagnosis: Sens: 70.6; Spec: 61.3; Acc: 68.5 Local diagnosis: Sens: 96.1; Spec: 20.8; Acc: 79.0	mrAI had a lesser performance than the centralized and local diagnosis in T-staging.
Peng w et al., 2024 [28]	60/117	NR	NR	Overall -T-staging accuracy FSEDL vs. Reader 3: 70 vs. 58.33; p = 0.016 FSEDL vs. Reader 1: 76.7 vs. 60; p = 0.021	FSEDL provided improved T-staging accuracy than FSE.
Tian C et al., 2023 [23]	317	265 (83.6)	52 (16.4)	AUC (SE): 0.902 (0.735); Sensitivity (SE): 0.75 (0.68); Specificity (SE): 0.96 (0.79); Accuracy (SE): 0.85 (0.73); PLR (SE): 18.0 (3.26); NLR (SE): 0.26 (0.41); PPV (SE): 0.95 (0.79); NPV (SE): 0.77 (0.68); NRI: 0.238	DL-based automatic models have a good performance in T-stage assessment.
Wan L et al., 2023 [19]	611	444 (73)	Validation: 81 (13); Test: 86 (14	AUC Range of eight DL models: 0.80 (0.75-0.85) to 0.89 (0.85-0.92); Training set: 0.77 (0.62-0.92); Validation set: 0.89 (0.76-1.00) AUC: AI model vs. pooled readers: 0.79 (0.70-0.89) vs. 0.54 (0.48-0.60) p < 0.001)	The DL model performed better radiologists in predicting lymph node metastasis in patients with stage T1-2 RC.
Ma S et al., 2023 [20]	253	152(60%)	101(40%)	Validation set 1 AUC: Nomogram: 0.908 (0.834 to 0.956); Radscore: 0.735 (.638 to 0.818); Subj Eva: 0.64 (0.539 to 0.733); Sensitivity:Nomogram:81.8%; Radscore:50.0%; Subj Eva: 86.4%; Specificity:Nomogram:94.9%; Radscore:92.4%; Subj Eva: 45.6%; Accuracy: Nomogram:92.1%; Radscore:83.2%; Subj Eva: 54.5%; PLR: Nomogram: 16.159; Radscore: 6.583; Subj Eva: 1.670; NLR: Nomogram:0.191; Radscore:0.541; Subj Eva:0.199; PPV: Nomogram:0.818; Radscore:0.647; Subj Eva:0.317; NPV: Nomogram:0.949; Radscore:0.869; Subj Eva:0.947 ; Performance (Nomogram vs. Radscore: 0<0.001; Nomogram vs. Subj Eva: 0<0.001) Validation set 2 AUC: Nomogram: 0.884(0.798-0.943); Radscore: 0.802 (0.704-0.879); Subj Eva: 0.734 (0.629-0.823); Sensitivity: Nomogram: 66.7%; Radscore: 66.7%; Subj Eva: 83.3%; Specificity: Nomogram: 96.9%; Radscore: 92.2%; Subj Eva: 62.5%; Accuracy: Nomogram: 88.6%; Radscore: 85.2%; Subj Eva:71.6%; PLR: Nomogram: 21.33; Radscore: 8.533; Subj Eva: 2.222; NLR: Nomogram: 0.344; Radscore: 0.362; Subj Eva: 0.267; PPV: Nomogram: 0.889; Radscore: 0.762; Subj Eva: 0.455; NPV: Nomogram: 0.886; Radscore: 0.881; Subj Eva:0.909; Performance (Nomogram vs. Radscore: 0.035; Nomogram vs. Subj Eva: 0.018)	DL-based radiomic nomogram had high performance and Radscore and subjective evaluation.
Wei Y et al., 2023 [21]	260	208 (80)	52 (20)	AUC AI-model :0.854; Radiologist's assessment: 0.678; clinical model: 0.747; T2W-model: 0.735; DWI-model: 0.759; DCE-model: 0.789	The multiparametric DL model had higher performance than the radiologist's assessment, the clinical model as well as the single parameter models in T-staging of RC patients.
Hou M et al., 2022 [18]	706; T1/2: 287 T3/4:419	565 (80)	141 (20)	SUPER Resolution T2 AUC: 0.869 (0.813–0.925); Sens: 0.711 (0.627–0.795); Spec: 0.931 (0.917–0.945); Accuracy: 0.833 (0.779–0.887) High Resolution T2 AUC: 0.810 (0.734–0.886); Sens: 0.895 (0.831–0.959); Spec: 0.701 (0.604–0.798); Accuracy: 0.773 (0.672–0.874) DLRS vs. expert radiologists (T1/2 and T3/4) AUC = 0.685, 95% CI 0.595–0.775	SUPER Resolution T2 appeared to have a better performance than the high-resolution T2 and expert radiologists
Yuan Y et al, 2022 [22]	204 (Cohort 1: 60; Cohort 2: 144)	NR	Test: 230; Validation: 94	AUC Cohort 1: Without rectal filling: 0.806; With rectal filling: 0.946 Cohort 2: Without rectal filling: 0.783; With rectal filling: 0.920 Without rectal filling vs. With rectal filling (p = 0.021)	The radiomics model with rectal filling showed better performance than the model without filling.
Liu X et al., 2021 [17]	235; Primary: 170; Validation: 65	162 (68.9)	62 (31.1)	C-index Primary cohort Nomogram: 0.865 (0.814-0.916); Clinical: 0.714 (0.632-0.797); DLRS: 0.851 (0.795-0.906); T2W: 0.854 (0.802-0.905); ADC: 0.688 (0.615-0.761). Validation cohort Nomogram: 0.775 (0.695-0.856); Clinical: 0.601 (0.487-0.7320; DLRS: 0.747 (0.665-0.830); T2W: 0.729 (0.640-0.819); ADC: 0.599 (0.473-0.724) NRI Primary cohort: Nomogram vs. Clinical: 0.599 (0.501-0.697; p =<0.001); DLRS vs. Clinical: 0.529 (0.406-0.651; p<0.001) Validation cohort: Nomogram vs. Clinical: 0.395 (0.195-0.595; p<0.001); DLRS vs. Clinical: 0.28 (0.089-0.483; p = 0.004) IDI. Primary cohort: Nomogram vs. Clinical: 0.409(0.310-0.508; p<0.001); DLRS vs. Clinical: 0.384 (0.275-0.493; p<0.001) Validation cohort: Nomogram vs. Clinical: 0.260 (0.117-0.402; p< 0.001); DLRS vs. Clinical: 0.187 (0.023-0.352; p = 0.026)	Nomogram had the better performance in the primary and validation cohorts.
Wu QY et al., 2021 [26]	183	146 (80)	37 (20)	AUC Overall Coronal plane: 0.98; Horizontal plane: 0.99; Sagittal plane: 0.97 AUC for different stages Coronal plane: T1: 0.96; T2: 0.97; T3: 0.97; T4: 0.97 Horizontal plane: T1: 1; T2: 1; T3: 1; T41 Sagittal plane: T1: 0.95; T2: 0.99; T3: 0.96; T4: 1.00. Accuracy AI: 100; Manual: 86	R-CNN AI is more effective than the manual method.
Wen DG et al., 2021 [29]	131	111 (85)	37 (15)	Training set: Sens: 0.810; Spec: 0.875; AUC: 0.893; Accuracy: 0.838 Test set: Sens: 0.810; Spec: 0.813; AUC: 0.810; Accuracy: 0.811 Original dataset: Sens: 0.810; Spec: 0.830; AUC:0.860	The automated ML model showed a better performance.
Zhao X et al., 2019 [24]	374	293 (78.3)	Internal: 31 (8.3) External: 50 (13.4)	Internal Dataset: 3T2WI: Sen:63 (59.7-65.9); PPV:54.7 (51.7-57.7); FP/vol: 15.7(13.5-18.0); DSC: 0.85(0.84-0.86) 3DWI: Sens: 52.0 (48.7-55.2); PPV: 66.7 (63.1-70.1); FP/vol: 7.8 (6.2-9.5); DSC: 0.63 (0.62-0.65) 2T2WI+1DWI: Sens: 81.3 (78.6-83.7); PPV: 59.7 (56.9-62.4); FP/Vol: 16.5 (14.1-19.0); DSC: 0.83 (0.82-0.84) 2DWI+1T2WI Sens: 80.0 (76.9-82.2); PPV: 73.5 (70.7-76.2); FP/vol: 8.6 (6.9-10.3); DSC: 0.82 (0.81-0.83) External Dataset 3T2WI: Sens: 45.5 (42.7-48.4); PPV: 44.2 (41.4-47.0); FP/vol: 13.8 (12.2-15.4); DSC: 0.85 (0.85-0.86) 3DWI: Sens: 36.0 (33.4-38.7); PPV: 44.7 (41.7-47.7); FP/Vol: 11.9 (9.9-13.8); DSC: 0.56 (0.54-0.57) 2T2WI+1DWI: Sens: 58.1 (55.2-60.9); PPV: 56.0 (53.2-58.7); FP/Vol: 11.0 (9.1-12.9); DSC: 0.84 (0.84-0.85) 2DWI+1T2WI: Sens: 62.6 (59.5-65.1); PPV: 64.5 (61.7-67.3); FP/vol: 8.2 (7.0-9.5); DSC: 0.81 (0.80-0.82)	DL-based LNDS model can facilitate N-staging in clinical practice.
He B et al., 2019 [27]	118	89 (75%)	29 (15%)	AUC Training set: Grade 1: 0.918; Grade 2: 0.822; Grade 3: 0.775; Grade 4: 1.000 Testing set: Grade 1: 0.717; Grade 2: 0.683; Grade 3: 0.690; Grade 4: 0.827 Specificity Training set: Grade 1: 0.800; Grade 2: 0.769; Grade 3: 0.706; Grade 4: 1.000 Testing set: Grade 1: 0.789; Grade 2: 0.875; Grade 3: 0.718; Grade 4: 1.000 Sensitivity Training set: Grade 1: 0.941; Grade 2: 0.846; Grade 3: 0.773; Grade 4: 1.000 Testing set: Grade 1: 0.650; Grade 2: 0.508; Grade 3: 0.650; Grade 4: 0.649	Radiomics feature AI models showed an acceptable performance in RC grading.

Role of AI in MRI-Based RC Staging

In a study by Hamabe et al. [25], an MRI-assisted AI algorithm for T-stage prediction in RC was compared with radiologists and pathologists using retrospective data from 69 institutions. The AI model achieved a sensitivity of 70.6%, specificity of 61.3%, and accuracy of 68.5%, which was lower than both centralized diagnosis (sensitivity: 84.2%, specificity: 37.7%, accuracy: 73.7%) and local diagnosis (sensitivity: 96.1%, specificity: 20.8%, accuracy: 79.0%). These results indicate that while the mrAI model showed reasonable performance, it lagged behind centralized and local diagnostic approaches in the T-staging of RC.

Wu et al. [26] used a DNN using a Faster R-CNN to predict preoperative T-staging of RC using MRI data from 183 patients. The AI model demonstrated exceptional performance, with AUC values of 0.98, 0.99, and 0.97 for the coronal, horizontal, and sagittal planes, respectively. For specific T-stages, the model achieved AUCs ranging from 0.95 to 1.00 across the different planes. The AI model also had higher performance than manual methods, achieving 100% accuracy compared to 86% for manual assessment. These results indicate that the R-CNN-based AI model is more effective than manual methods for T-staging prediction in RC.

T2-Weighted MRI Image-Based AI Models

A study by Xia et al. [30] evaluated the performance of the WISDOM model in staging lymph node involvement using T2W MRI images for N-staging. The study compared the model’s performance to that of senior and junior radiologists, both with and without AI assistance. The WISDOM model achieved an AUC of 0.78 (95% CI: 0.73-0.82) for binary staging and 0.80 (95% CI: 0.76-0.84) for ternary staging, significantly higher performance than junior radiologists (AUC 0.69, 95% CI: 0.64-0.73) and showing a marked improvement when combined with AI (AUC 0.80, P < 0.001). Senior radiologists alone had an AUC of 0.792 (95% CI: 75.1-83.0), which increased to 0.88 (95% CI: 0.85-0.91) with AI assistance (P < 0.001). In terms of the C-index, the WISDOM model also showed superior performance (MI: 0.73, MIS: 0.74, MISA: 0.76) with significant improvements when AI was added to both junior and senior radiologists (P < 0.001 and P = 0.007, respectively). These findings indicate that the WISDOM model performed better than human radiologists and that combining AI with radiologists resulted in improved performance for both staging accuracy and consistency.

A study by Tian et al. [23] used CNN DL models evaluated for differentiating T-stages of RC using high-resolution T2-weighted (HR-T2W) MRI images. The study included 317 patients, with 265 (83.6%) diagnosed with early-stage and 52 (16.4%) with advanced-stage RC. The DL-based automatic model achieved an AUC of 0.902 (SE: 0.735), demonstrating high diagnostic performance. Additional metrics included a sensitivity of 0.75 (SE: 0.68), specificity of 0.96 (SE: 0.79), and accuracy of 0.85 (SE: 0.73). The positive likelihood ratio (PLR) was 18.0 (SE: 3.26), while the negative likelihood ratio (NLR) was 0.26 (SE: 0.41). The model showed a positive predictive value (PPV) of 0.95 (SE: 0.79), a negative predictive value (NPV) of 0.77 (SE: 0.68), and a net reclassification improvement (NRI) of 0.238. These findings suggest that DL-based automatic models exhibit strong performance in accurately assessing the T-stage in RC, which was more than subjective evaluation.

In a study by Wan et al. [19], DL models based on four 2D and 3D ResNet architectures (ResNet18, ResNet50, ResNet101, and ResNet152) were evaluated for predicting lymph node metastasis in stage T1-2 RC patients using T2-weighted MRI. The DL models showed AUC values ranging from 0.80 to 0.89, with the validation set achieving an AUC of 0.89 (0.76-1.00). Compared to pooled radiologists, the DL model is significantly higher than human readers (AUC: 0.79 vs. 0.54, p < 0.001). These results highlight the superior predictive capability of the DL model for lymph node metastasis in early-stage RC.

Yuan et al. [22] developed a DL radiomics model to improve T-staging accuracy in RC patients using HR-T2WI MRI. The model with rectal filling had a better performance than that one without filling, achieving higher AUC values in both cohorts. In Cohort 1, the AUC was 0.946 with rectal filling, compared to 0.806 without filling, and in Cohort 2, the AUC was 0.920 with rectal filling versus 0.783 without. The results (p = 0.021) indicate that the radiomics model with rectal filling significantly improves T-staging accuracy.

Spin Echo MRI Image-Based AI Models

In a prospective study by Peng et al. [28], the performance of DLR-based FSE MRI for T- and N-staging in RC was compared to standard FSE MRI in 60 patients. The study found that the DLR-based FSE MRI (FSEDL) significantly improved T-staging accuracy compared to radiologist assessments. Specifically, FSEDL achieved a T-staging accuracy of 70%, while Reader 3 reached 58.33% (p = 0.016), and Reader 1 achieved 60% compared to 76.7% for FSEDL (p = 0.021). These results demonstrate that DLR-based FSE MRI provides better T-staging accuracy than standard FSE MRI.

In a study by He et al. [27], a radiomics-based RF model was developed for MRI-based tumour grading in RC using axial T2-weighted turbo spin echo images without fat suppression. The model showed strong performance in the training set, with AUC values ranging from 0.775 for Grade 3 to 1.000 for Grade 4. In the testing set, AUC values ranged from 0.683 for Grade 2 to 0.827 for Grade 4. Although sensitivity and specificity varied across grades, the radiomics AI model demonstrated acceptable performance for tumour staging in RC.

Combined MRI Image-Based AI Models

In a study by Zhao et al. [24], a DL framework using Mask R-CNN was applied to detect lymph nodes and perform N-staging in RC using fused T2WI and DWI MRI images. The model was evaluated on both internal and external datasets from six hospitals, showing promising results. For the internal dataset, the model achieved sensitivity up to 81.3% (2T2WI+1DWI), with a Dice Similarity Coefficient (DSC) of 0.83, while for the external dataset, sensitivity reached 62.6% (2DWI+1T2WI) with a DSC of 0.81. The DL model also demonstrated strong performance in terms of other performance metrics, more than radiologists in several metrics. These findings suggest that the DL-based lymph node detection and staging (LNDS) model could significantly enhance N-staging in clinical practice.

In a study by Liu et al. [17], the performance of a DL-based radiomics score (DLRS) was evaluated for predicting metastasis-based T-staging in locally advanced rectal cancer (LARC) using multiparametric MRI (T2WI and DWI). In the primary cohort, the nomogram achieved the highest C-index (0.865), followed by DLRS (0.851), while clinical assessment showed the lowest (0.714). In the validation cohort, the nomogram also more performed DLRS and clinical assessments in both the C-index and net reclassification index (NRI). Overall, the nomogram demonstrated superior performance in both cohorts, than that of DLRS and manual methods for predicting T-staging in LARC patients.

Hou et al. [18] developed a DL-based 3D super-resolution (DL-3D SR) MRI radiomics model that was evaluated for preoperative T-staging in RC using a combination of multiple MRI sequences. The DL-3D SR model achieved an AUC of 0.869, sensitivity of 0.711, specificity of 0.931, and accuracy of 0.833, which was more than both high-resolution T2 (AUC = 0.810) and expert radiologists (AUC = 0.685) for T1/2 and T3/4 staging. These results suggest that the DL-3D SR model provides superior performance compared to traditional high-resolution T2 MRI and expert assessments for T-staging in RC.

A DL-based clinical-radiomics nomogram was developed by Ma et al. [20] for N-stage prediction in RC patients using axial DWI, T2WI, T1WI, and HR-T2WI. The nomogram demonstrated superior performance with an AUC of 0.908 in the validation set, compared to Radscore (AUC = 0.735) and subjective evaluation (AUC = 0.640). The nomogram also achieved higher sensitivity (81.8%), specificity (94.9%), accuracy (92.1%), and PPV (0.818) than both Radscore and subjective evaluation. These results highlight the nomogram's effectiveness through AI in predicting N-stage in RC patients, higher than the traditional methods.

Wei et al. [21] developed a DL model utilizing multiparametric CNN to improve T-staging accuracy in RC patients using dynamic contrast-enhanced T2WI and DWI. The AI model achieved an AUC of 0.854, which was higher than the radiologist's assessment (AUC = 0.678), clinical model (AUC = 0.747), and single-parameter models (T2WI = 0.735, DWI = 0.759, DCE = 0.789). These findings demonstrate that the multiparametric DL model significantly enhances T-staging accuracy compared to traditional methods.

In a study by Wen et al. [29], an automated ML model was developed to predict preoperative T-staging in RC patients using T2WI and RS-EPI DWI images. The model demonstrated strong performance in both the training and test sets, with an AUC of 0.893 and accuracy of 0.838 in the training set and an AUC of 0.810 and accuracy of 0.811 in the test set. Overall, the automated ML model is better than conventional methods, showing consistent sensitivity (0.810) and specificity (0.830). These results suggest that the ML model is more effective for T-staging prediction in RC patients.

Discussion

This systematic review assessed the performance of MRI-based AI models in RC staging, focusing on the various imaging techniques and AI methodologies employed across the studies. RC staging is a critical step in determining treatment strategy and prognosis, and MRI, with its ability to provide high-resolution imaging, has become a key modality in this process [10]. The integration of AI, particularly DL techniques, has shown promise in enhancing the accuracy and efficiency of staging, as evidenced by the findings from the included studies.

The studies in this review utilized multiple imaging modalities [31]. These MRI sequences provide complementary information on tumour characteristics, tissue differentiation, and lesion boundaries, making them ideal for training AI models in RC staging [13]. The combination of these imaging modalities in several studies, including the use of FSE sequences, likely contributed to the high performance observed in many of the AI models [32].

The use of various advanced DL techniques, such as CNN, DNN, and R-CNN, reflects the rapid evolution of AI methods in medical imaging. These methods, particularly CNN and DNN, are well-suited to image classification tasks due to their ability to automatically extract relevant features from complex datasets. In addition, the use of frameworks like WISDOM and multiparametric CNN models in some studies suggests that there is growing interest in developing more sophisticated models that can leverage both image data and clinical/radiological features, further enhancing model performance [33]. The application of clinical-radiomics nomograms and hybrid approaches such as DL-based radiomics models also signals a promising direction in personalized medicine, where AI is combined with patient-specific data for more accurate staging predictions [34].

Multiple performance metrics were used to assess the clinical usefulness of these AI models.

Sensitivity and specificity are particularly important in staging, as high sensitivity ensures that the model can accurately identify cases of advanced-stage cancer, which is critical for treatment planning, while high specificity reduces the likelihood of false positives, which could lead to over-treatment [35]. The studies included in this review generally reported strong performance across these metrics, with some models achieving sensitivity and specificity values exceeding 90%. In addition, the C-index was also a useful measure of model performance, providing an overall evaluation of how well the models rank patients based on the predicted likelihood of having a particular cancer stage.

A notable aspect of the studies included in this review is the data split used for model training and testing. Most studies used 70-80% of the dataset for training and 20-30% for testing the model, which is a common approach in AI research. However, two studies, those by Xia et al. [30] and Ma et al. [20] used only 60% of their data for training, which may have implications for the robustness and generalizability of their models. Smaller training datasets may limit the model's ability to generalize well to unseen data, which is an important consideration when developing AI tools for clinical applications. The relatively smaller training datasets in these studies could be a potential limitation [36], and future work might benefit from using larger, more diverse datasets to enhance model generalizability and reduce overfitting.

Limitations and Future Directions

Despite the promising results from these studies, there are several limitations that warrant attention. First, while the use of multiple MRI modalities helps capture a wide range of imaging features, it also increases the complexity of model development and may contribute to issues related to data heterogeneity. Standardization of imaging protocols across different centres would be essential to ensure that AI models are generalizable across diverse clinical settings. The pathology reference standard of each individual LN detected at MRI was not available, which is to be stratified in future studies.

In addition, many of the AI models reviewed were developed and validated on relatively small datasets and from one country, which raises concerns about their scalability and real-world applicability. Although the use of cross-validation and external validation cohorts in some studies helps mitigate these concerns, larger, multi-centre studies are necessary to confirm the findings and assess the generalizability of the models.

Another limitation is the lack of consistent reporting on model interpretability. While DL models, particularly CNNs, often achieve high performance, they are frequently described as "black boxes," meaning their decision-making processes are not always transparent. This lack of interpretability can hinder clinical adoption, as healthcare professionals may be hesitant to trust models that do not provide clear explanations for their predictions. Future studies should focus on improving model transparency and incorporating explainable AI methods to enhance clinician confidence in using these tools.

Finally, the integration of AI models into clinical workflows remains a significant challenge. Even with promising performance metrics, successful implementation of AI-based systems in clinical practice requires careful consideration of factors such as workflow integration, clinician training, and regulatory approval. Future research should focus not only on improving model accuracy but also on developing frameworks for the seamless integration of AI tools into routine clinical practice.

## Conclusions

The current systematic review indicates that MRI-based AI models for RC staging show great promise, with several studies demonstrating high performance across key metrics like sensitivity, specificity, accuracy, and AUC. However, smaller dataset sizes, model generalizability, and clinical integration remain challenges in implementation. Future research should focus on expanding datasets, improving model interpretability, and exploring real-world implementation strategies to fully realize the clinical potential of AI in RC staging.

## Appendices

Appendix A

**Table 3:** Search strategy

Search number	Query	Results
1	"Artificial intelligence"[Title/Abstract]	61,669
2	"Machine learning"[Title/Abstract]	1,29,600
3	AI[Title/Abstract]	70,925
4	ML[Title/Abstract]	4,55,818
5	"Deep learning"[Title/Abstract]	73,936
6	#1 OR #2 OR #3 OR #4 OR #5	7,02,478
7	"Magnetic Resonance Imaging"[Mesh]	5,60,925
8	MRI[Title/Abstract]	3,54,416
9	"Magnetic resonance imaging"[Title/Abstract]	3,24,843
10	"Magnetic resonance*"[Title/Abstract]	4,72,945
11	#7 OR #8 OR #9 OR #10	8,62,228
12	"Rectal Cancer"[Title/Abstract]	31,827
13	"Rectal neoplasm"[Title/Abstract]	229
14	Rectal*[Title/Abstract]	1,10,151
15	#12 OR #13 OR #14	1,10,151
16	#6 AND #11 AND #15	448

Search number	Query	Results
1	‘Artificial intelligence’ OR ‘Machine learning’ OR ‘Deep learning’	332800
2	‘magnetic resonance imaging’	1272730
3	‘rectum cancer’	48156
4	#1 AND #2 AND #3	

Appendix B

**Table 4:** Reason for exclusion of studies

No	Title	Reason for exclusion
1	Multiparametric MRI-based machine learning models for preoperatively predicting rectal adenoma with canceration	Not rectal cancer staging
2	Magnetic resonance imaging-based deep learning model to predict multiple firings in double-stapled colorectal anastomosis	Not rectal cancer staging
3	Preoperative prediction of tumor deposits in rectal cancer with clinical-magnetic resonance deep learning-based radiomic models	Not rectal cancer staging
4	Multiparameter MRI-based radiomics for preoperative prediction of extramural venous invasion in rectal cancer	Not rectal cancer staging
5	A deep learning nomogram kit for predicting metastatic lymph nodes in rectal cancer	Not rectal cancer staging
6	Evaluation of Rectal Cancer Circumferential Resection Margin Using Faster Region-Based Convolutional Neural Network in High-Resolution Magnetic Resonance Images	Not rectal cancer staging
7	Region-specific deep learning models for accurate segmentation of rectal structures on post-chemoradiation T2w MRI: a multi-institutional, multi-reader study	Not rectal cancer staging
8	Deep transfer learning based on magnetic resonance imaging can improve the diagnosis of lymph node metastasis in patients with rectal cancer	Not rectal cancer staging
9	Preoperative Grading of Rectal Cancer with Multiple DWI Models, DWI-Derived Biological Markers, and Machine Learning Classifiers	Not rectal cancer staging
10	Preoperative Prediction of Microsatellite Instability in Rectal Cancer Using Five Machine Learning Algorithms Based on Multiparametric MRI Radiomics	Not rectal cancer staging
11	Preoperative prediction of tumor budding in rectal cancer using multiple machine learning algorithms based on MRI T2WI radiomics	Not rectal cancer staging
12	Predicting rectal cancer tumor budding grading based on MRI and CT with multimodal deep transfer learning: A dual-center study	Not rectal cancer staging
13	Evaluation of Deep Learning Clinical Target Volumes Auto-Contouring for Magnetic Resonance Imaging-Guided Online Adaptive Treatment of Rectal Cancer	Not rectal cancer staging
14	Predictive Study of Machine Learning-Based Multiparametric MRI Radiomics Nomogram for Perineural Invasion in Rectal Cancer: A Pilot Study	Not rectal cancer staging
15	Response of locally advanced rectal cancer (LARC) to radiochemotherapy: DW-MRI and multiparametric PET/CT in correlation with histopathology	Not rectal cancer staging
16	Multiparametric MRI-based radiomic model for predicting lymph node metastasis after neoadjuvant chemoradiotherapy in locally advanced rectal cancer	Not rectal cancer staging
17	Artificial intelligence-based technology for semi-automated segmentation of rectal cancer using high-resolution MRI	Cancer segmentation
18	MRI-based automatic segmentation of rectal cancer using 2D U-Net on two independent cohorts	Cancer segmentation
19	Technical Note: A deep learning-based autosegmentation of rectal tumors in MR images	Cancer segmentation
20	Deep Learning for Fully-Automated Localization and Segmentation of Rectal Cancer on Multiparametric MR	Cancer segmentation
21	Improved deep learning for automatic localisation and segmentation of rectal cancer on T2-weighted MRI	Cancer segmentation
22	Automatic segmentation of rectal tumor on diffusion-weighted images by deep learning with U-Net	Cancer segmentation
23	Segmentation of rectal tumor from multi-parametric MRI images using an attention-based fusion network	Cancer segmentation
24	Dual parallel net: A novel deep learning model for rectal tumor segmentation via CNN and transformer with Gaussian Mixture prior	Cancer segmentation
25	A fully automatic deep learning algorithm to segment rectal Cancer on MR images: a multi-center study	Cancer segmentation
26	Deep Learning Algorithm‑Based MRI Radiomics and Pathomics for Predicting Microsatellite Instability Status in Rectal Cancer: A Multicenter Study	Cancer segmentation
27	Imaging segmentation mechanism for rectal tumors using improved U-Net	Cancer segmentation
28	Fully semantic segmentation for rectal cancer based on post-nCRT MRl modality and deep learning framework	Cancer segmentation
29	Comparable Performance of Deep Learning-Based to Manual-Based Tumor Segmentation in KRAS/NRAS/BRAF Mutation Prediction With MR-Based Radiomics in Rectal Cancer	Cancer segmentation
30	Incorporating patient-specific information for the development of rectal tumor auto-segmentation models for online adaptive magnetic resonance Image-guided radiotherapy	Cancer segmentation
31	A medical image segmentation method for rectal tumors based on multi-scale feature retention and multiple attention mechanisms	Cancer segmentation
32	Predicting the prognosis of lower rectal cancer using preoperative magnetic resonance imaging with artificial intelligence	Not outcome of interest
33	Dynamic Contrast-enhanced Magnetic Resonance Imaging Evaluation of Whole Tumour Perfusion Heterogeneity Predicts Distant Disease-free Survival in Locally Advanced Rectal Cancer	Not outcome of interest
34	Value of texture analysis based on dynamic contrast-enhanced magnetic resonance imaging in preoperative assessment of extramural venous invasion in rectal cancer	Not outcome of interest
35	Towards deep-learning (DL) based fully automated target delineation for rectal cancer neoadjuvant radiotherapy using a divide-and-conquer strategy: a study with multicenter blind and randomized validation	Not outcome of interest
36	Radiomics Based on T2-Weighted Imaging and Apparent Diffusion Coefficient Images for Preoperative Evaluation of Lymph Node Metastasis in Rectal Cancer Patients	Not outcome of interest
37	Artificial intelligence system of faster region-based convolutional neural network surpassing senior radiologists in evaluation of metastatic lymph nodes of rectal cancer	Not outcome of interest
38	Improved U-Net based on contour prediction for efficient segmentation of rectal cancer	Not MRI-based AI
39	MRI-directed high-frequency (29MhZ) TRUS-guided biopsies: initial results of a single-center study	Not MRI-based AI
40	Preoperative prediction of tumour deposits in rectal cancer by an artificial neural network-based US radiomics model	Not MRI-based AI
41	Accuracy of high-resolution MRI with lumen distention in rectal cancer staging and circumferential margin involvement prediction	Not MRI-based AI
42	Radiomic Texture and Shape Descriptors of the Rectal Environment on Post-Chemoradiation T2-Weighted MRI are Associated with Pathologic Tumor Stage Regression in Rectal Cancers: A Retrospective, Multi-Institution Study	Not MRI-based AI
43	Risk factors and development of machine learning diagnostic models for lateral lymph node metastasis in rectal cancer: multicentre study	Not MRI-based AI
